# Chia Oil and Mucilage Nanoemulsion: Potential Strategy to Protect a Functional Ingredient

**DOI:** 10.3390/ijms24087384

**Published:** 2023-04-17

**Authors:** Sibele Santos Fernandes, Mariana Buranelo Egea, Myriam de las Mercedes Salas-Mellado, Maira Rubi Segura-Campos

**Affiliations:** 1 School of Chemistry and Food, Federal University of Rio Grande, Av Italy km 8, Carreiros 96203-900, Brazil; mysame@yahoo.com.br; 2 Goiano Federal Institute of Education, Science and Technology, Campus Rio Verde, Sul Goiana, Km 01, Rio Verde 75901-970, Brazil; 3 Faculty of Chemical Engineering, Autonomous University of Yucatán, Periférico Norte km 33.5, Tablaje Catastral 13615, Mexico; maira.segura@correo.uady.mx

**Keywords:** chia seed, omega 3, nanoencapsulation, chia mucilage

## Abstract

Nanoencapsulation can increase the stability of bioactive compounds, ensuring protection against physical, chemical, or biological degradations, and allows to control of the release of these biocompounds. Chia oil is rich in polyunsaturated fatty acids—8% corresponds to omega 3 and 19% to omega 6—resulting in high susceptibility to oxidation. Encapsulation techniques allow the addition of chia oil to food to maintain its functionality. In this sense, one strategy is to use the nanoemulsion technique to protect chia oil from degradation. Therefore, this review aims to present the state-of-the-art use of nanoemulsion as a new encapsulation approach to chia oil. Furthermore, the chia mucilage—another chia seed product—is an excellent material for encapsulation due to its good emulsification properties (capacity and stability), solubility, and water and oil retention capacities. Currently, most studies of chia oil focus on microencapsulation, with few studies involving nanoencapsulation. Chia oil nanoemulsion using chia mucilage presents itself as a strategy for adding chia oil to foods, guaranteeing the functionality and oxidative stability of this oil.

## 1. Introduction

The use of nanotechnology in food production has great potential to modernize the food process and its characteristics, provide new foods and processing methods, as well as revolutionize how food reaches the consumer [1]. The encapsulation, a nanotechnology technique, of bioactive compounds has represented a viable and efficient alternative to increase physical-chemical stability, protect from interactions with other food ingredients, maintain bioactivity, and control the release of these compounds [2,3].

Emulsions gain great prominence among nanoencapsulation techniques and can be characterized as (i) microemulsion, which is generally used to refer to a substance that is thermodynamically stable and composed of a mixture of oil, water, and surfactants, and (ii) nanoemulsion that is considered a conventional emulsion that contains very small particles [4].

There are many hydrophobic or poorly soluble nutrients and bioactive compounds that are essential for human health, such as water-insoluble vitamins, phenolic acids, fatty acids, and essential oils, among others. Applying these ingredients directly to food can have several limiting factors, including low stability due to low sensitivity to oxygen, light, and temperature, as well as low solubility and ability to withstand the conditions of gastrointestinal transit to be absorbed and perform this ingredient function in the human body [5,6]. Among the ingredients that demonstrate great instability and, simultaneously, the potential to be included in foods are the fatty acids, mainly the polyunsaturated fatty acid, such as omega 3. These compounds have a high degree of unsaturation in the molecule, resulting in high susceptibility to oxidation [7].

**Figure 1:**
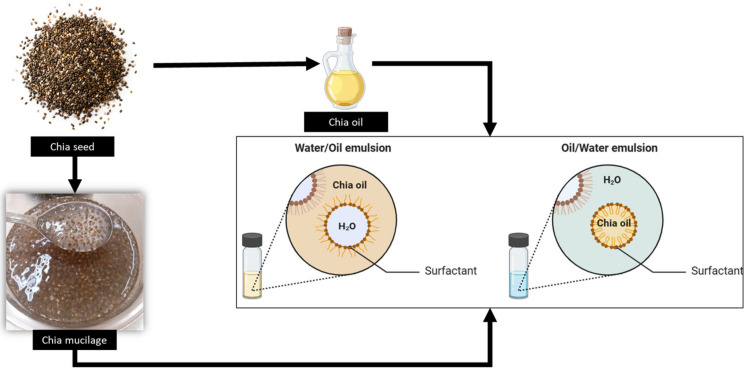
Scheme demonstrating chia seed and its products (oil and mucilage) and emulsion formation from them.

An oil that stands out for its higher quantity and quality of unsaturated fatty acids than other plant sources is chia seed oil [8]. Chia (Figure 1) is a seed that has been consumed for thousands of years. Currently, research has associated this consumption with health-promoting properties and high nutritional value, especially polyunsaturated fatty acids, dietary fiber, proteins, vitamins, etc. [9,10].

Another ingredient that can be extracted from chia or its by-products is mucilage, which becomes more evident when the seed is placed in contact with water and a transparent mucilaginous gel is obtained [11]. Chia mucilage contains ~72% soluble fiber, in addition to monosaccharides, mainly arabinose and xylose, followed by glucose, fructose, galactose, rhamnose, and mannose [12,13]. 

The excellent technological properties of chia mucilage are due to its composition and chemical structure, guaranteeing great potential for use in the food, pharmaceutical, and packaging industries [14]. Chia mucilage is used as a fat substitute [15,16,17,18], texturizing agent [19,20], film-forming agent [21,22,23,24,25], cosmetic and pharmaceutical ingredient [26], and effluent treatment [27]. In addition, it can be used as a wall material in micro [28] and nanoencapsulation [29] techniques.

Investigating and evaluating new techniques that are easy to develop and cost-effective and that, in addition, allow chia oil encapsulation to be efficient when using new polymers, such as chia mucilage, is of paramount importance [30]. Therefore, this review aims to present the state-of-the-art use of different encapsulation methods as a protection strategy for chia oil and the use of chia mucilage as its potential carrier.

## 2. Chia Seeds: An Oil and Mucilage Ingredient Source

Chia seed contains approximately 10–19% protein [9,15,31]; 29–33% lipids composed mainly of unsaturated fatty acids, such as linolenic (58–60%), linoleic (19–21%), and oleic (9–11%) acids, and others in smaller amounts and saturated acids, such as palmitic (6–8%) [31], and 34–40% of dietary fiber [9]. In addition, chia seed stands out for its mineral content, such as magnesium, potassium, and phosphorus [9], and phenolic compounds, such as myricetin and rosmarinic, 3,4-dihydroxybenzoic, and caffeic acids (>30 mg/100 g), along with others in smaller quantities [31].

**Figure 2:**
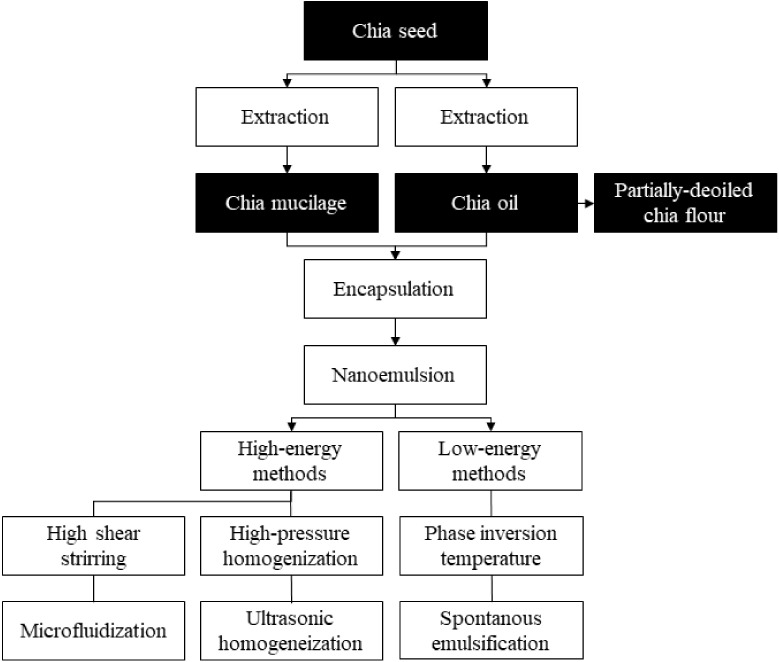
Summary flowchart of oil and mucilage separation from chia (*Salvia hispanica* L.) seed.

Two important components can be extracted from chia seeds: (i) oil and (ii) mucilage (Figure 2). The (i) chia oil is extracted using a cold press, solvent, and supercritical CO_2_ techniques, which influence the yield and composition of the final product. Extraction using supercritical provides a higher extraction yield and higher levels of linoleic and linolenic acid [32]. While obtaining via pressing is time-consuming, the oil shelf life and phytochemical composition depend on the operating conditions, such as pressure, temperature, and time [33]. The choice of method must be associated with its subsequent application, such as food or pharmaceutical [11].

Chia seed contains oil (~20%), which in turn is rich in polyunsaturated fatty acid (PUFA), α-linolenic [ALA; 18:3 (omega 3)] (~68%), and linoleic acid [LA; 18:2 (omega 6)] (~19%) [32], showing a highly beneficial omega 3/omega 6 fatty acid proportion [34]. Also, chia oil contains small amounts of oleic, palmitic, and stearic acids, as well as other bioactive components, such as tocopherols, polyphenols, carotenoids, and phospholipids [35]. 

In addition, a by-product resulting from the extraction of chia oil, called partially-deoiled chia flour, contains approximately 27% of protein, 59% of dietary fiber, 222 mg gallic acid/100 g of total polyphenols, and its high content of omega 3 (~6900 mg/100 g) fatty acids and a high omega 3/omega 6 proportion (~3.2 ratios) [34]. 

The proven benefits of ingesting chia oil are based on a significant increase in plasma linolenic acid content [36,37,38,39], a significant increase in levels of eicosapentaenoic acid (EPA) [38,39,40], control of hyperglycemia and reduction of systolic blood pressure in diabetics [41,42,43], antioxidant potential [44,45], anti-inflammatory activity [46], and the induction of the browning process in subcutaneous white adipose tissue (WAT) [47].

The incorporation of chia seed oil in food, due to its nutritional properties, is useful for the prevention of cardiovascular diseases and the maintenance of human health. However, the high degree of unsaturation of the compounds present in this oil (omega 3 and its ratio with omega 6) implies the need to use a process that allows their incorporation into food, eliminating the susceptibility to oxidation and the development of off-flavors that affect the sensory properties of food [48]. The technique that has been most used for this purpose is encapsulation [49].

Meanwhile, (ii) mucilage is a hydrocolloid composed of long-chain, high-molecular-weight polymers, together with polysaccharides and proteins with high affinity for water that are partially or completely soluble, where they disperse and form viscous solutions [50]. This functional ingredient with a high amount of dietary fiber is mainly extracted using hydration, extraction, and recovery steps, often in water [51]. Although the yield of the chia mucilage extraction process ranges from 2–6%, this ingredient contains ~10–26% protein [13,15] and 76–79% of carbohydrates, mainly arabinose (41–52%) and xylose (35–44%) [13]. The literature has related the composition of saccharides to the antioxidant capacity presented by this mucilage [52]. Ali et al. [53] proposed the structure of chia mucilage as a tetrasaccharide with 4-O-methyl-α-D-glucuronopyranosyl residues with β-D-xylopyranosyl branches in the main chain.

Mucilage is a potential replacer for fat/oil, egg, and gluten and an emulsifier/stabilizer in various foods, such as baked goods, dairy, cereal, and meat products. The market for this ingredient has grown increasingly with the new niche of plant-based consumers (flexitarians, vegans, and vegetarians, among others) [54]. In addition, chia mucilage can be used as an encapsulating/stabilizing agent for chia oil (as discussed in Section 3.3).

## 3. Encapsulation

Encapsulation is a process in which the oil droplets are surrounded by a coating or incorporated in a homogeneous or heterogeneous matrix to obtain small capsules with different properties. This matrix isolates the bioactive molecule from the environment until its release in response to external conditions, such as pH, pressure, and temperature, among others [55].

Many attractive micronutrients nutritionally used for food fortification cannot be added simply to the product. Therefore, the nanoencapsulation of hydrophilic carriers is an alternative to increase the solubility and bioavailability of these compounds. In addition to protecting unstable compounds against unfavorable conditions during processing, storage, and transportation, nanoencapsulation can improve the bioactivity of the compounds [5,55]. Besides, the stability, bioavailability, and bioefficacy of the active compounds depend largely on the food matrix and the encapsulation method used [55].

The choice of the encapsulation method, as well as the selection of the wall material for a specific application, depends on the required particle size, physical and chemical properties of the core and the wall, application of the final product, desired mechanisms release, production scale, and process cost [56]. In this sense, among the innumerable encapsulation types, nanoemulsions stand out due to the small size of the particles, which avoids instability processes when applied to food. 

Nanoemulsions are colloidal delivery systems commonly used to encapsulate bioactive lipophilic compounds, such as PUFAs, essential oils, carotenoids, and stilbenes. Nanoemulsion technology can provide new applications for oils to extend shelf life or add nutritional value to foods [57]. In addition, the nanoemulsion technique solves problems of the low solubility of lipophilic compounds in water, easy oxidation, and difficulty absorbing oil-soluble functional components [58]. 

The use of chia oil nanoencapsulation techniques remains a poorly studied field that deserves more attention since the association of the advantages of using encapsulation by nanoemulsions to preserve chia oil has high potential [59]. 

### 3.1. Nanoemulsion

Nanoemulsion, also called miniemulsion, submicron emulsion, ultrafine emulsion, or dispersed emulsion. This emulsion type consists of a very fine dispersion composed of an oily phase (such as triglycerides or hydrocarbons) and an aqueous phase (water or water with some electrolyte or polyol), showing higher stability than microemulsions [60,61,62].

Nanoemulsions can be of the oil-in-water type if the oil is dispersed as droplets in the water (o/w) or water-in-oil if the water is dispersed as droplets in the oil (w/o) (Figure 2). The structure of the particles in a nanoemulsion is similar to that found in a microemulsion, as the nonpolar part of the surfactant molecules projects into the hydrophobic core formed by the oil, while the polar part of the surfactant molecules projects to the surrounding water phase [4]. 

**Figure 3:**
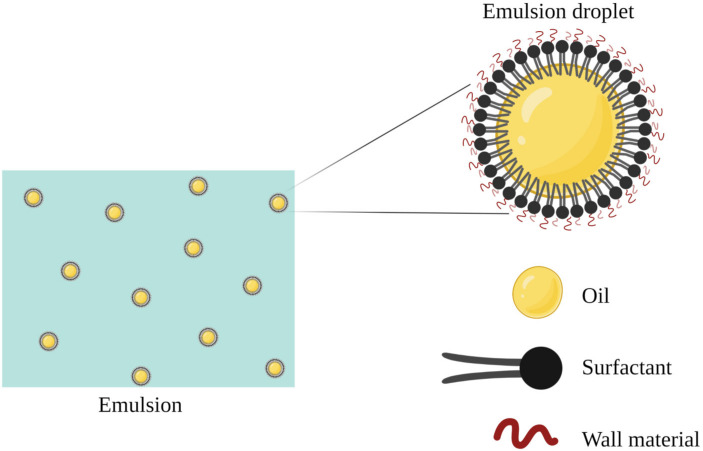
Scheme representing the structure of emulsions and their stability in systems.

A similar structure of microemulsions and nanoemulsions produced from oil, water, and surfactant is demonstrated in Figure 3, showing a hydrophobic core of oil and surfactant and a hydrophilic coating.

Some authors related that the size of nanoemulsions ranges from 10 to 1000 nm [63] and 20 to 200 nm [64], while others that the droplet diameter is less than 100 nm [60], demonstrating that there is no consensus in the literature. Nanoemulsions are most frequently used to encapsulate essential oils, such as cinnamon [65], and also in antimicrobial delivery systems [66]. 

Other components can be added to the nanoemulsion formulations. The addition of solvents is a simple method that supports the preparation of nanoemulsions where water is insoluble in some bioactive compounds because it allows the preparation of nanodispersions in a step with low energy consumption with high encapsulation performance [67]. When the organic phase, formed for an organic solvent that is miscible in water and contains lipophilic bioactive substances, is added to the aqueous phase containing an emulsifier, nanoparticles will instantly form at the interface between the organic and aqueous phases by rapid diffusion of the organic solvent into the aqueous phase. The boundary layers of the organic solvent form the places of maximum overlap of the bioactive compound, nucleation, and growth of the particles occur. Therefore, the affinity of the emulsifier towards bioactive compounds is crucial for the formation of nanoparticles and for avoiding their aggregation [68,69]. 

The bioactive compound to be encapsulated is stabilized by one emulsifier or a combination of emulsifiers. There are a wide variety of emulsifiers used in the food industry, but milk proteins are among the most important emulsifiers used up to now [67,70].

The nanoemulsions have various benefits, such as physical stability and the bioavailability of encapsulated active substances, which are melted, also avoiding conventional destabilization phenomena, such as creams, sedimentation, coalescence, and flocculation [71]. Nanoemulsion has long-term stability as it can prevent precipitation and coalescence. The main reason for this stability is due to the small particle size, which causes the effects of Brownian motion to dominate the gravitational force, neutralizing the kinetic instability caused by gravity or viscosity. Also, nanoemulsion presents good stability against aggregation since the band of attractive forces that act between the particles decreases with the decrease in size, while the steric repulsion band is less dependent on the size of the particles [72,73].

### 3.2. Nanoemulsion Preparation Methods

Nanoemulsions do not form spontaneously, requiring energy to enter the system, and therefore, the methods for nanoemulsion production are classified as those that require high and low energy [57]. High-energy methods use mechanical devices capable of generating intense shear forces that can break structures on the order of micrometers into nanometric particles. Low-energy methods are based on the spontaneous formation of oil droplets in mixed oil-water-surfactant systems when the solution or environmental conditions are altered using the chemical energy of the system [62,72,74].

High-energy methods have several advantages, including high efficiency, good availability to scale up, and the possibility of using various types of emulsifiers and oil [73]. These methods involve high-shear stirring, microfluidization, high-pressure homogenization, or ultrasonic homogenization, while the low-energy ones rely on spontaneous emulsification and phase inversion. 

Table 1 presents all methods already used to form chia oil nanoemulsions. Notably, the number of studies involving chia oil nanoemulsion is still limited. Most of the studies are focused on using microencapsulation techniques, showing that using new chia oil nanoencapsulation techniques, more precisely the nanoemulsion, is a promising area of study.

#### 3.2.1. Methods That Use High-Energy

Of the available methods, the choice of which to use depends on the desired mean droplet size, which is directly linked to the type of homogenizer; its operating conditions, such as energy intensity, time, and temperature; composition of the sample, such as type of oil and surfactant and their respective concentrations; as well as the physical-chemical properties of the sample such as viscosity and interfacial tension [79,80].

Regarding high-energy methods, only the (i) high shear stirring technique was used to produce chia oil nanoemulsion [75,76,77]. Studies using (ii) high-pressure homogenization, (iii) microfluidization, and (iv) ultrasonic homogenization techniques for the nanoencapsulation of chia oil were not found in the literature, revealing a potential field for future study.

The (i) high-shear stirring can be used as a preliminary technique for preparing nanoemulsions [58,70,81]. High-speed rotor-based devices, such as UltraTurrax, a high-performance dispersing machine, when compared to other high-energy methods, do not provide good dispersion in terms of particle size in addition to dissipating energy in the form of heat [82].

Campo et al. [75] developed chia seed oil nanoparticles using an UltraTurrax machine, and they determined the nanoparticle’s stability during storage under accelerated conditions. The authors obtained particle sizes from 201 to 209 nm, an encapsulation efficiency of 82.8%, and high thermal stability, as well as an improvement in the oxidative stability of the oil during storage. 

Fernandes et al. [76] studied the development of chia oil nanoemulsions by varying the concentrations of the bioactive compound and the encapsulating material using the UltraTurrax machine at different agitation speeds, as well as the use of ethanol. These authors obtained an encapsulation efficiency between 88.8–97.3% and a particle size between 160.5–637.3 nm, with an excellent percentage of stability and storage stability at different temperatures.

Maldonado et al. [60] evaluated avocado, linseed, or chia oils in the formulation of nanoemulsions enriched with α-tocopherol prepared using the UltraTurrax machine. Chia and linseed nanoemulsions demonstrated small particle sizes (124 and 122 nm, respectively). The nanoemulsion developed from avocado oil showed the highest oxidative stability compared with chia and linseed oils due to its composition with more monounsaturated fatty acids.

The (ii) high-pressure homogenization can be performed under cold and hot temperatures, providing ideal conditions for scaling up [83]. High-pressure homogenizers work with pressures between 50 and 100 MPa and are widely used to form nanoemulsions [84].

In the literature, some studies compare high-pressure homogenization with other nanoemulsion formation processes. Kotta et al. [85] used Capryol 90 (propylene glycol monocaprylate) and Transcutol HP (diethylene glycol monoethyl ether) in the oils phase and polysorbate 20 (Tween 20) as a surfactant to compare high-pressure homogenization and phase inversion methods. The authors concluded that high-pressure homogenization produced nanoemulsions in almost all experiments, even with 8% surfactant, but the polydispersity index was considered high. Furthermore, the authors mentioned that the low-energy method produced efficient and more uniform nanoemulsions when compared to the high-energy method. In this same sense, Yukuyama et al. [86] determined the conditions that produce olive oil nanoemulsions prepared through high-pressure homogenization and phase emulsification as high- and low-energy processes, respectively.

Zhao et al. [52] developed lycopene nanoemulsions with sesame, walnut, and linseed oils through the homogenization process and using lactoferrin as an emulsifier. The authors verified that the sesame oil in nanoemulsions ─with lower viscosity, higher density, and lower unsaturation─ demonstrated high stability and bioaccessibility of lycopene compared with the other evaluated oils.

The (iii) microfluidization is a technique used for nanoemulsion preparation that applies a high pressure of 20,000 psi to generate high energy [87]. In this technique, an emulsion passes through an interaction chamber using a high-pressure pump device where there are flow channels, which in turn are designed to cause the emulsion currents to collide with one another at high speed, creating very high-pressure action and producing an exceptionally fine emulsion [88].

Komaiko, Sastrosubroto, and McClements [89] developed nanoemulsions enriched with omega 3 from fish oil through microfluidization using different types of natural sunflower phospholipids. These authors obtained nanoemulsions with particles smaller than 150 nm and zeta potential, mostly tending to negative. El-Messery et al. [57,58] produced krill oil nanoemulsions by combining three different biopolymers ─whey protein concentrate, maltodextrin, and gum arabic─ through microfluidization. Nanoemulsions with up to 8% krill oil showed good stability with a droplet diameter variation of 153.9 to 162.3 nm. Afterward, the authors dehydrated the nanoemulsion by spray-drying, and though the particle size of the nanoemulsions increased by at least 7 times after spray drying, the nanoemulsions demonstrated high bioaccessibility.

Teng et al. [59] developed chia oil nanoemulsions with polysorbate 80 (Tween 80) and sorbitan monooleate (Span 80), sodium caseinate, and sucrose monopalmitate through microfluidization (9000 psi to 17,000 psi, 6 passes). The nanoemulsions presented particle size from 100 to 200 nm and showed good stability when stored at room temperature or 4 °C for two weeks. In addition, the authors used the nanoemulsion composed of Tween 80 and Span 80 (0.5% by weight emulsifier and 15,000 psi six times) to evaluate its stability in an application at different temperatures and in real food samples. This nanoemulsion was relatively stable after heating at 95 °C for different times based on the mean particle diameter and polydispersion index.

The (iv) ultrasonic emulsification uses a probe that generates ultrasonic waves to disintegrate the macroemulsion by cavitation forces. Its main advantages are that it is an easy, fast, low-cost, clean (no solvents are necessary) method and uses high energy [88].

This process occurs through two types of mechanisms. The first mechanism consists of an acoustic field that generates a combination of interfacial waves. The instability caused leads to the eruption of the oil phase in an aqueous medium in the form of droplets. The second mechanism consists of low-frequency ultrasound waves that decay the droplets by cavitation near the interface, generating extreme instability of primary droplets producing nanoemulsion with very small droplet size [90,91].

The type and amount of surfactant and homogenization time influence emulsification. Ultrasound should not be used in excess since degradation of some components present in the nanoemulsion may occur due to the high energy applied [92]. An option to reduce the size of the particles is to associate more than one technique, such as using an UltraTurrax machine followed by ultrasound methods [93].

Branco, Sen, and Rinaldi [94] studied the effect of sodium alginate in different types of oil (corn oil and oleic acid) on the quality of nanoemulsions produced by ultrasound homogenization. This method produced a nanoemulsion of oil-in-water and polysaccharide systems with satisfactory physicochemical properties.

#### 3.2.2. Methods That Use Low-Energy

Compared to the high-energy method, the low-energy methods have great advantages due to the simplicity of the flotation of nanoemulsions and because it does not require expensive or sophisticated manufacturing equipment [95].

Among the low-energy methods, such as (i) phase inversion temperature and (ii) spontaneous emulsification, chia oil nanoemulsion was only produced using spontaneous emulsification [59,78]. However, the phase inversion temperature technique also seems to be promising for developing chia oil nanoemulsions since there are recent works producing cajeput essential oil nanoemulsions [96]. 

The (i) phase inversion temperature (PIT) method is a relatively simple and fast way to prepare nanoemulsions with small droplet sizes and narrow size distribution. The principle of this method is to heat surfactant, oil, and water to a temperature above or near the phase inversion and then rapidly cool with continuous stirring to spontaneously form fine oil droplets. The temperature of the phase inversion is identified because the turbidity of the system decreases significantly due to the formation of a bicontinuous microemulsion or lamellar structure that does not strongly diffuse light [97,98].

A disadvantage of the PIT method for certain types of oil is the heating of the emulsions, which can cause thermal degradation and loss of volatility of active ingredients [97]. This technique is being widely used for the preparation of antimicrobial cinnamon oil nanoemulsions [98,99]. Due to the disadvantage, the antimicrobial activity of nanoemulsions could be reduced. Therefore, using a lower PIT (but not very low as nanoemulsions can physically break due to accelerated droplet coalescence) during nanoemulsion preparation could avoid thermal excess degradation [99]. In addition to antimicrobial activity, this technique produces nanoemulsions with antifungal activity [100].

Although the PIT method has received much more attention than high-energy emulsification methods because it generates smaller and more uniform droplets without requiring sophisticated devices, it is still not used much in studying polyunsaturated fatty acid materials (such as chia oil) due to the aforementioned disadvantage.

The (ii) spontaneous emulsification has already been used for the development of nanoemulsions with antimicrobial activity, such as nanoemulsions of cinnamon oil [101], and to encapsulate the bioactive compounds present in fish [102], lemon, fish, grapeseed, roasted sesame, canola, peanut, and extra virgin olive oils [103].

Although using expensive and sophisticated equipment is not necessary, the main disadvantage of the spontaneous emulsion method is that it requires high levels of synthetic surfactants, which is undesirable for many food applications due to high cost, taste, and regulatory issues. However, this technology is still useful for applications where small amounts of lipophilic components must be incorporated into clear water-based products, such as flavors, nutraceuticals, vitamins, or antimicrobials [103].

Liew et al. [71] produced lime essential oil nanoemulsions from key lime (*Citrus aurantifolia*), kaffir lime (*Citrus hystrix*), and calamansi lime (*Citrofortunella microcarpa*) by the spontaneous emulsification method. These authors concluded that the lime nanoemulsions showed great potential to be incorporated into water-based food products and beverages as a flavoring and antimicrobial agent.

Besides the microfluidization technique, Teng et al. [59] studied chia seed oil nanoemulsions using spontaneous emulsification with Tween 80 and Span 80, sodium caseinate, or sucrose monoesters as emulsifiers. Nanoemulsions prepared through spontaneous emulsification presented particle sizes between 150 to 200 nm. Only chia seed oil with Tween 80 and Span 80 could be produced by spontaneous emulsification, suggesting that the microfluidization method has a wider application range than spontaneous emulsification for polyunsaturated fatty acids.

Kaya, Oztop, and Alpas [78] developed chia oil nanoemulsions with different surfactant concentrations (1, 2, 2.5, 2.75, 3 e 4, *w*/*w*) using spontaneous emulsification followed by high-pressure homogenization. The authors obtained nanoemulsions with droplet sizes varying from 90 to 2850 nm. Droplets showed a more complex multilayer phase structure, and high-pressure homogenization accelerated aggregation and coalescence of droplet size, and as pressure increased, average droplet size also increased. Nanoemulsion stability was 97–98%, representing a strong, stable condition.

### 3.3. Wall Material for Chia Oil Nanoemulsion

Oil nanocapsules can be produced with different wall materials, depending on the use and the type of oil nanoencapsulation. In general, the wall material is used alone because the particles formed must have a size of 1000 nm, and, as there is a greater surface contact and fewer compounds in the formation of the nanocapsule, the greater the interaction between the compounds, favoring in the last analysis the size of the particles [104]. Most oil nanoemulsion studies use alginate [73,94], polycaprolactone [105], maltodextrin [57], and whey protein concentrate [106] as wall material. 

Currently, many researchers have focused their studies on finding wall materials that can improve the individual retention and protection characteristics of encapsulated active compounds [76]. Mucilages have been studied as a wall material for nanoparticles due to their high retention capacity of bioactive compounds and ease of chemical modifications to improve their stability [107]. In this sense, many authors have proven the efficiency of different mucilage sources as a wall material to produce nanoparticles of bioactive compounds, such as vitamins, minerals, fatty acids, and flavorings [108,109].

Among these, chia mucilage has been gaining prominence [76]. Cortés-Camargo et al. [110] developed lemon essential oil microcapsules prepared using mixtures of mesquite gum and chia mucilage. Antigo et al. [111] evaluated the effect of chia mucilage as a microencapsulating agent for beet betacyanin. Dehghani et al. [29] evaluated the potential of chia mucilage in developing green cardamom essential oil nanofibers. This demonstrates the potential of using chia mucilage as a wall material for chia oil. All authors cited above found that due to the high viscosity, high water-holding capacity, and emulsifying properties of chia mucilage, higher encapsulation efficiencies, smaller particle sizes, and storage stability were obtained. Furthermore, the authors found that chia mucilage may be a potential nanocarrier for antibacterial and antioxidant deliveries.

Chia mucilage is a transparent mucilaginous gel that is obtained when the chia seed is immersed in water and is essentially composed of soluble fibers [53]. This has already been used as a substitute for fat, as it can hydrate, develop viscosity, and preserve freshness, especially for bakery products [15]. It was also used as a substitute for emulsifiers and stabilizers in ice cream [112]. Dick et al. [113], Munõz et al. [114,115], and Fernandes et al. [22] used chia mucilage as a film-forming agent. The films exhibited acceptable tensile strength, as well as extensibility and flexibility.

Regarding the microencapsulation of chia oil, chia mucilage has already been used as wall material through ionic gelation [116] and spray drying [117,118]. Regarding the nanoencapsulation of chia oil, there are still few studies.

Campo et al. [75] and Fernandes et al. [76] described a chia oil nanoemulsion using chia mucilage as the encapsulating material. The authors obtained promising results for protection action using chia mucilage. The high encapsulation efficiency found by the authors (>90%) [78,79] may have been caused by the high emulsifying effect of chia mucilage, about 63.7% [16]. Associated with this, chia mucilage, as an encapsulating agent, forms a network with the active material, reducing solubility [117].

In that same context, Stefani et al. [119] also developed linseed oil nanoemulsions using chia mucilage as a wall material. The authors obtained an encapsulation efficiency of 52% and a particle size of 356 nm. However, all the authors verified that the chia mucilage showed excellent properties for acting as an encapsulating agent.

## 4. Conclusions

This review presents the use of chia oil nanoemulsion as a strategy to protect this oil. Studies on chia oil nanoencapsulation revealed that this is still a little-explored area. Allied with this deficit, the high degree of unsaturation of the essential fatty acids present in the chia oil (in higher concentrations than any other vegetable source) requires that some process be carried out offering protection from them so they can be added to food. Therefore, the study of the formation of nanoemulsions of chia oil is extremely important.

Studies in the literature on wall material, or as a substitute in food, suggest that chia mucilage can be used as a structuring material for nanoencapsulation compounds, mainly chia oil, allowing high solubility in food and facilitating the incorporation of nanoparticles in food.

## Figures and Tables

**Table 1:** Studies on the production of chia oil nanoemulsions.

Method	Technique	Components	Authors
HIGH-ENERGY	High shear stirring	Chia mucilage, Tween 80, and ethanol	[75]
		Chia mucilage, Tween 20, and ethanol	[76]
		α-tocopherol, soy lecithin, and Tween 80	[77]
	Microfluidization	Tween 80, Span 80, sodium caseinate, and sucrose monopalmitate were used as emulsifiers; highly hydrolyzed lecithin, polyglycerol ester, glycerin, propylene glycol, and sorbitol solution	[59]
LOW-ENERGY	Spontaneous emulsification		
HIGH-ENERGY + LOW-ENERGY	Spontaneous emulsification + High-pressure homogenization	Tween 80	[78]

## Data Availability

Not applicable because the paper is the opinion based on the analysis of the published literature.
